# Causal association of peripheral immune cell counts and atrial fibrillation: A Mendelian randomization study

**DOI:** 10.3389/fcvm.2022.1042938

**Published:** 2023-01-06

**Authors:** Yuntao Feng, Xuebo Liu, Hongwei Tan

**Affiliations:** Department of Cardiology, Tongji Hospital, Tongji University School of Medicine, Shanghai, China

**Keywords:** peripheral immunity, atrial fibrillation, Mendelian randomization, CD4 + lymphocytes, causation analysis

## Abstract

**Background:**

Atrial fibrillation (AF) is the most common and persistent form of arrhythmia. Recently, increasing evidence has shown a link between immune responses and atrial fibrillation. However, whether the immune response is a cause or consequence of AF remains unknown. We aimed to determine whether genetically predicted peripheral immunity might have a causal effect on AF.

**Methods:**

First, we performed Mendelian randomization (MR) analyses using genetic variants strongly associated with neutrophil, eosinophil, basophil, lymphocyte, and monocyte cell counts as instrumental variables (IVs). Lymphocyte counts were then subjected to further subgroup analysis. The effect of immune cell counts on AF risk was measured using summary statistics from genome-wide association studies (GWAS).

**Results:**

Two-sample MR analysis revealed that a higher neutrophil count, basophil count and lymphocyte count had a causal effect on AF [Odds ratio (OR), 1.06, 95% confidence interval (CI), 1.01–1.10, *P* = 0.0070; OR, 1.10; 95% CI, 1.04–1.17; *P* = 0.0015; OR, 0.96; 95% CI, 0.93–0.99; *P* = 0.0359]. In addition, in our further analysis, genetically predicted increases in CD4 + T-cell counts were also associated with an increased risk of AF (OR, 1.04; 95% CI, 1.0–.09; *P* = 0.0493).

**Conclusion:**

Our MR analysis provided evidence of a genetically predicted causal relationship between higher peripheral immune cell counts and AF. Subgroup analysis revealed the key role of peripheral lymphocytes in AF, especially the causal relationship between CD4 + T cell count and AF. These findings are beneficial for future exploration of the mechanism of AF.

## 1. Introduction

Atrial fibrillation (AF) is the most common and persistent form of arrhythmia and one of the most important factors leading to increased mortality. Approximately 46.3 million people suffer from AF globally (1). In addition, the burden of AF is expected to increase by more than 60% over the next 30 years and become one of the most significant epidemic and public health challenges (2).

Electrical remodeling, structural remodeling, and autonomic nerve remodeling are the pathological basis of AF; the immune system is known to play an important role in this process (3, 4). Inflammation mediated by immune cells in the myocardium is known to contribute to AF, thereby causing a cycle of malignant progression of atrial remodeling, inducing AF and increasing thrombosis (5). Furthermore, peripheral immunity is also correlated with AF (6). In order to uncover the association between white blood cell counts and AF, a subset of the Framingham Heart Study and a Norwegian study that followed 936 eligible participants for up to 5 years found that higher white blood cell (WBC) counts in peripheral immune cells were associated with an increased risk of AF (7). In addition, a high neutrophil-to-lymphocyte ratio (NLR) in peripheral immune cells was associated with an increased risk of new-onset AF in 21,118 subjects (8).

Although these previous clinical studies have revealed an association between peripheral immunity and AF, granulocyte counts in peripheral blood, particularly neutrophils, have been associated with an increased incidence of other cardiovascular diseases, such as hypertension, coronary heart disease and diabetes (9–11). However, the presence of these common risk factors may have caused bias. Furthermore, observational analysis may not remove potential confounders and unmeasured reverse causality (12). In addition, large-scale randomized clinical trials are expensive. Mendelian randomization (MR) analysis is a method that has been mainly used for etiological inference in epidemiology over recent years. Associations between exposure-related genetic variants and outcomes can represent the effect of exposure on outcomes (13). Since genetic variation is randomly assigned at conception, this effect is not affected by confounding factors and reverse causality, thus, providing another method for inferring causality (14). MR is a tool for analyzing causality between exposure and outcome using genetic variation as instrumental variants (IVs) (15).

Therefore, in this study, we selected single nucleotide polymorphism (SNP) data from large genome-wide association studies (GWAS) of hematologic traits as instrumental variables of exposure to assess the causal relationship between WBC counts and AF through a MR approach.

## 2. Materials and methods

### 2.1. Data sources

Peripheral blood cell counts and AF candidate genetic instruments (SNPs) were selected from previous genome-wide association studies (GWAS). To prevent pleiotropic bias in cross-lineage cases (16), all individuals in the study were of European ancestry. Peripheral blood cell counts, including neutrophils, lymphocytes, monocytes, neutrophils, eosinophils, and basophils were obtained from the Blood Cell Consortium meta-analysis, which includes data from 563,085 individuals of European ancestry (17). For further cell subpopulation analysis, including absolute cell counts for T-cell subtypes and B-cell subtypes, we used GWAS summary statistics for 3,757 individuals analyzed by flow cytometry (18). The genetic association dataset for AF was derived from a large meta-analysis of six discovery cohorts (The Nord-Trøndelag Health Study (HUNT), deCODE, the Michigan Genomics Initiative (MGI), DiscovEHR, UK Biobank, and the AFGen Consortium), including 1,030,836 subjects of European ancestry, which were divided into 60,620 AF cases and 970,216 controls (19) (Table 1).

**TABLE 1:** Description of included trails in the study.

Contribution	Traits	Sample size	Number of SNPs	Author	Population	GWAS ID
Exposure	Neutrophil cell count	5,63,946	–	Vuckovic et al.	European	ieu-b-34
	Basophil cell count	5,63,085	–	Vuckovic et al.	European	ieu-b-29
	Lymphocyte cell count	5,63,085	–	Vuckovic et al.	European	ieu-b-32
	Monocyte cell count	5,63,085	–	Vuckovic et al.	European	ieu-b-31
	Eosinophil cell count	5,63,085	–	Vuckovic et al.	European	ieu-b-33
	HLA DR + Natural Killer Absolute Count	3,580	1,51,58,016	Valeria Orrù et al.	European	ebi-a-GCST90001648
	Natural Killer T AC	3,653	1,51,95,758	Valeria Orrù et al.	European	ebi-a-GCST90001621
	CD4 + CD8dim AC	3,652	1,51,95,743	Valeria Orrù et al.	European	ebi-a-GCST90001609
	CD8 + AC	3,652	1,51,95,743	Valeria Orrù et al.	European	ebi-a-GCST90001592
	Resting Treg AC	3,405	1,51,31,843	Valeria Orrù et al.	European	ebi-a-GCST90001480
	Secreting Treg AC	3,405	1,51,31,843	Valeria Orrù et al.	European	ebi-a-GCST90001492
	B cell AC	3,653	1,51,95,758	Valeria Orrù et al.	European	ebi-a-GCST90001642
	Unswitched memory B cell AC	3,656	1,50,48,937	Valeria Orrù et al.	European	ebi-a-GCST90001398
Outcome	Atrial fibrillation	10,30,836	10,30,836	Nielsen JB et al.	European	ebi-a-GCST006414

AC, absolute count.

### 2.2. Selection of genetic IVs

Three key assumptions needed to be met in the study design: (1) IVs were significantly correlated with interest exposure; (2) IVs were not associated with any confounders of the exposure-outcome association; and (3) IVs impact outcomes only through exposure (20).

To meet these conditions, we first set parameters for identifying IVs, including a *P*-value of 5 × 10^–8^ for genome-wide significance, a linkage disequilibrium clustering algorithm with an *R*^2^ threshold = 0.001 over the 10 Kb region to ensure the independence of IVs exposure and allowing a minor allele frequency of 0.3 for SNPs in the palindromic region. Statistics relating to the association between these genetic variants and AF were then extracted as an outcome with a more relaxed aggregation threshold (*R*^2^ < 0.01). Following identification, Phenoscanner (21) was used to remove SNPs that may have violated the second and third key hypothesis and which may have a pleiotropic effect on other phenotypes (body mass index, smoking status, hypertension, coronary artery disease, chronic renal failure, and diabetes). Finally, pleiotropic outliers were identified and excluded with MR pleiotropy residual sum and outlier (MR-PRESSO) (22). A flow chart of our selection of IVs is given in Supplementary Figure 1 and summary information for the SNPs used for MR analyses are given in Supplementary Tables 1–6.

The proportion of variance explained (PVE) by each IV was used to explain the strength of the selected SNPs and was calculated as PVE = 2 × EAF × (1-EAF) × β^2^ (EAF, effect allele frequency; β, effect size on the exposure). Instrument strength was then assessed by the F-statistic which reflects the exposure variance explained by the instrument variables (23). Calculation of the F-statistic was based on PVE value *via* [PVE × (n – 1 – k)]/[(1 – PVE) × k], where n represented the effective sample size in the exposure GWAS, and k representsed the number of variants included in the IV model. To determine the power of MR outcomes, we use an online calculator^1^ to perform power estimation through a given type I error rate of alpha 0.05 and the OR from IVW estimates. A summary of information for the IVs used for MR analyses after clumping and data harmonization can be found in Supplementary Table 7.

### 2.3. Statistical analysis

The MR study was performed in R version 4.0.2 (The R Development Core Team, Vienna, Austria) using the TwoSampleMR (TSMR) R package version 0.5.5. TSMR analysis was used to determine the causal relationship between peripheral immunity and HF in which the inverse variance-weighted (IVW) method was used to estimate the causal relationship between exposure and results (24). Highest precision and unbiased causal estimates can be provided by IVW when there are no invalid genetic instrumental variables (25). Additional analyses were performed, including the weighted median method and the MR-PRESSO method (26) to avoid the bias of pleiotropic effects. To address the potential violation of the IV hypothesis, we applied constrained maximum likelihood and model averaging and the Bayesian Information Criterion (cML-MA-BIC) method (27). Potential directional pleiotropy was evaluated by MR-Egger regression intercept (28). All GWAS analyses were calibrated using the Bonferroni method. Leave-one-out (LOO) sensitivity analysis was then used to determine the association of individual SNPs and whether the results were driven by any single SNP (29). Finally, funnel plots and scatter plots were used to visually investigate heterogeneity (30, 31).

### 2.4. Data availability statement

The datasets processed in this study were derived from GWAS (17–19). GWAS data are publicly available abstract level data (32); thus, no ethical approval was required.

## 3. Results

### 3.1. Effects of genetically predicted peripheral blood cell counts on AF

**FIGURE 1:**
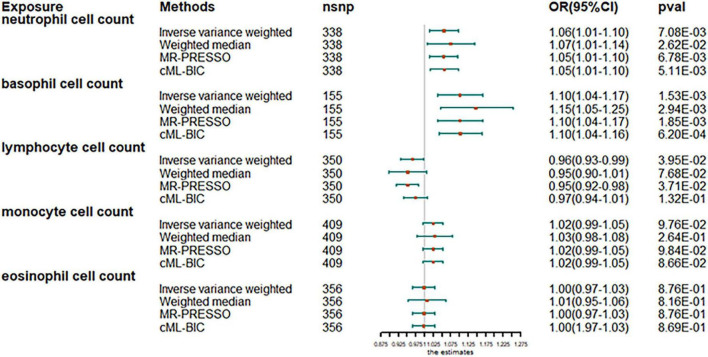
Mendelian randomization estimates of the association between blood cell counts and risk of atrial fibrillation. OR, odds ratio; CI, confidence interval.

The analysis after evaluation and removal of SNPs associated with confounding is shown in Figure 1. Since no significant heterogeneity was observed in the Cochran Q test, a fixed-effects model was used to estimate MR effect sizes. IVW (fixed effects) analysis showed that higher neutrophil counts were strongly associated with an increased susceptibility to AF [odds ratio (OR), 1.06; 95% confidence interval (CI), 1.01–1.10; *P* = 0.0070]. In addition, basophil counts were also observed to be associated with an increased susceptibility to AF in other leukocyte subtypes (OR, 1.10; 95% CI, 1.04–1.17; *P* = 0.0018). However, lymphocyte count was inversely associated with disease risk (OR, 0.97; 95% CI, 0.93–0.99; *P* = 0.0481), thus, suggesting the protective role of lymphocytes in AF. Sensitivity analyses showed no genetic polymorphism bias in any of the genetically predicted immune cell count analyses. LOO analysis further showed that the significance of the results was not driven by any single SNP. No obvious horizontal polytropism was found by visual inspection of funnel plots and analysis of MR-Egger regression intercepts. The results of the heterogeneity test, the polytropy test and the F-statistic are summarized in Supplementary Table 7. However, eosinophil or monocyte counts were not significantly associated with AF, although a positive trend was observed.

### 3.2. Causal effect of lymphocyte subtype on AF

**FIGURE 2:**
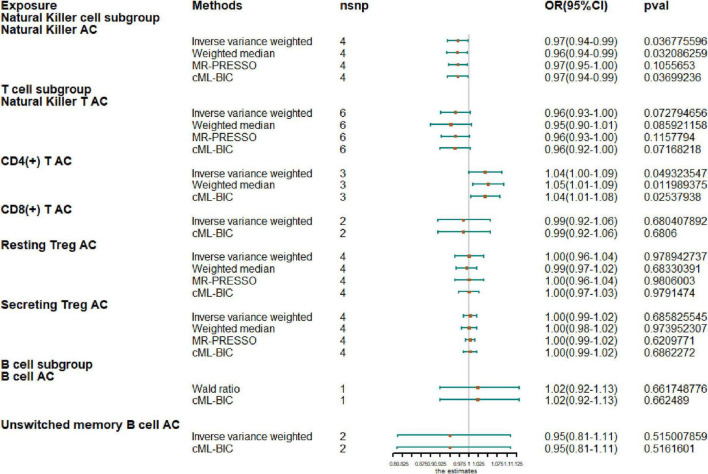
Mendelian randomization results for the relationship between cell counts of lymphocyte subpopulation and atrial fibrillation. AC, absolute count.

Next, we extended our analysis by further measuring causal estimates of the risk of AF by natural killer (NK), T and B cells in lymphocytes. Due to the small sample size of GWAS, we only evaluated eight lymphocyte subsets by MR, including NK cells, NKT cells, resting Tregs, secreting Tregs, CD4^+^ T cells, CD8^+^T cells, B cells, and unswitched memory B cells. If there were less than two IV variables available, Wald ratio results are shown instead of IVW and when there were fewer than three IVs, only IVW and CML-BIC are shown (Figure 2). Our analysis showed that increased CD4 + T-cell counts were associated with a higher risk of AF (OR, 1.04; 95% CI, 1.0–1.09; *P* = 0.0493). An increase of NK cell count was associated with a protective effect on AF (OR, 0.97; 95% CI, 0.94–0.99; *P* = 0.0368) and the NKT cell count was also negatively correlated with AF (OR, 0.96; 95% CI, 0.93–1.00; *P* = 0.0728), although the results were not significant. However, we also observed that genetically predicted increases in CD4 + T-cell counts were associated with a higher risk of AF (OR, 1.04; 95% CI, 1.0–1.09; *P* = 0.0493). In addition, due to the sample size, we did not observe a causal effect of other cell subtypes on AF risk and all results were supported by other MR methods.

## 4. Discussion

Recent studies have shown that considerable changes in the immune system occur during AF, including the recruitment and activation of immune cells and the secretion of their immune molecules induced by various factors; this is a process called immune remodeling (33). Immune remodeling runs through the entire process of the occurrence and maintenance of AF. This process does not only cause myocardial electrical, structural, and neural changes, but also induces AF-related pathological changes including fibrosis, thus playing an important role in AF (34). Furthermore, the results of observational studies indicate that immune cell-mediated atrial remodeling and inflammation are present in AF atria but not in non-AF atria (35). Therefore, the peripheral immune status of AF patients may also be different, thus, indicating the causal relationship between peripheral immunity and AF.

Neutrophils are an important component of peripheral immunity and can increase AF susceptibility by releasing cytokines, such as IL-6, TNF-α, and IL-1β (36). In addition, granulocytes are also the main source of reactive oxygen species (ROS) and myeloperoxidase (MPO), which can mediate AF by mediating oxidative stress (37). Here, we provide evidence of genetic causality between neutrophil count and AF, thus, suggesting an enhanced effect on AF. Peripheral eosinophils play an important role in inflammation and atrial remodeling in AF, and eosinophil products, such as major basic protein (MBP), can lead to endocardial fibrosis (38, 39). In addition, peripheral basophils play an important role in tissue fibrosis in heart allograft models. The depletion of basophils can inhibit the progression of allograft fibrosis (40). In the previous study, however, no association was found between eosinophils or basophils and AF (7). Our results elucidate the positive genetic causality between basophil count and AF. However, there is no evidence for a causal relationship between genetically predicted eosinophil counts and AF. More data from randomized clinical trials are still needed to support our hypothesis. Monocytes in the peripheral immune system can trigger an inflammatory cascade involving cytokine release and play an important role in fibrosis and heart failure; however, their role in AF remains unknown (41). Our results also cannot reveal the genetic causality between monocyte count and AF. More data from randomized clinical trials are now needed to prove this relationship. It has been reported that a low peripheral lymphocyte count is associated with inflammation and a reduction in lymphocyte count reflects the level of inflammation. In addition, a high level of NLR is also a risk predictor of AF (8, 42). Consistent with previous reports, we provide evidence of a negative genetic causal association between lymphocyte count and AF, thus, suggesting that lymphocytes may have a protective effect against AF.

Lymphocytes play different roles in AF and the combination of different roles of different lymphocytes in AF constitutes the protective effect of total lymphocyte count in AF. NK cells could alter the local cytokine environment by preventing the maturation and trafficking of inflammatory cells. In the myocardium, NK cells can prevent the development of cardiac fibrosis by limiting collagen formation in cardiac fibroblasts and by inhibiting the accumulation of specific inflammatory populations and profibrotic cells in the heart (43); this is consistent with our results in that NK cell counts have a protective effect on AF. NKT cells are mainly found in the liver and a core component of the immune response during liver fibrosis (44). Moreover, the adoptive transfer of NKT cells was also shown to protect mice from pulmonary fibrosis (45). In our experiment, it was observed that NKT cell count was negatively correlated with AF, although this was not significant. More experiments are needed to prove this point. CD4^+^ T can be activated by Toll-like receptor 2 (TLR2) and TLR4 to participate in the activation of AF (36). In response to antigens, co-stimulators, and cytokines, CD4^+^ T cells can differentiate into different subsets of helper T (Th) cells. TH1 cells promote macrophage efficacy and mediate inflammation in AF by secreting interferon-γ (44). In contrast, Th2 cells counteract the Th1 response by secreting several pro-fibrotic cytokines (46). Th17 cells can promote the development of AF by secreting IL-17A to promote inflammation and cardiac fibrosis (47). CD4 ^+^ CD25 ^+^ regulatory T cells (Tregs) have been recognized to play an important role in maintaining peripheral tolerance and limiting inflammatory disease; furthermore, the depletion of Tregs can aggravate myocardial fibrosis (48). Studies on the role of CD8^+^ T in AF are rarely reported (36). B lymphocytes play a role in the humoral immune component of the adaptive immune system mainly by secreting antibodies (49). The abnormal activation of B cells can produce pathological autoantibodies. Evidence suggests that autoimmunity can mediate cardiovascular disease and may be a possible mechanism for AF (50). To explore the relationship between lymphocyte subtypes and AF, we further analyzed several subtypes of B and T cells and found a genetic causal relationship between CD4^+^T cell counts and AF. However, our results do not indicate a causal relationship between genetically predicted other lymphocyte subtypes and AF, which means that more data from randomized clinical trials are still needed to explore their relationship.

In conclusion, we demonstrate a causal effect of peripheral immunity on AF based on MR results obtained from large-scale aggregated GWAS data. In addition, the protective effect of total lymphocyte cell count in AF may be synthesized by the protective and promotive effects in NK cells and CD4^+^ T cells. Our research enhances current understanding of the role of the peripheral immune system in AF. Further studies are now necessary to understand the relationship between different peripheral immune cells and AF and the underlying mechanisms.

## 5. Limitations

There are some limitations to our study that need to be considered. First, the results of other MR methods are not entirely consistent with IVW methods in univariate MR analysis. We cannot completely exclude the possibility of pleiotropy in peripheral immunity and AF. Second, our study mainly included participants of European descent, which cannot be generalized to other ethnic groups (51, 52). More data are now needed to be replicated in other populations. Third, we lack data on the quantity of peripheral immunity. Furthermore, we did not assess potential differences in the risk of AF among the orders of magnitude of peripheral immune cells. Fourth, lymphocyte subtype analysis based on small sample size may lead to insufficient power to detect the influence of lymphocyte subtype on AF.

## Data availability statement

The original contributions presented in this study are included in the article/Supplementary material, further inquiries can be directed to the corresponding author.

## Author contributions

YF designed the manuscript. HT and XL revised the manuscript. All authors contributed to the article and approved the submitted version.

## Footnotes

1
https://shiny.cnsgenomics.com/mRnd/
