# Supplementary figures and images for: Causal association of peripheral immune cell counts and atrial fibrillation: A Mendelian randomization study
Source: Front Cardiovasc Med. 2023 Jan 6;9:1042938. doi: 10.3389/fcvm.2022.1042938 (PMC9853293; doi:10.3389/fcvm.2022.1042938)

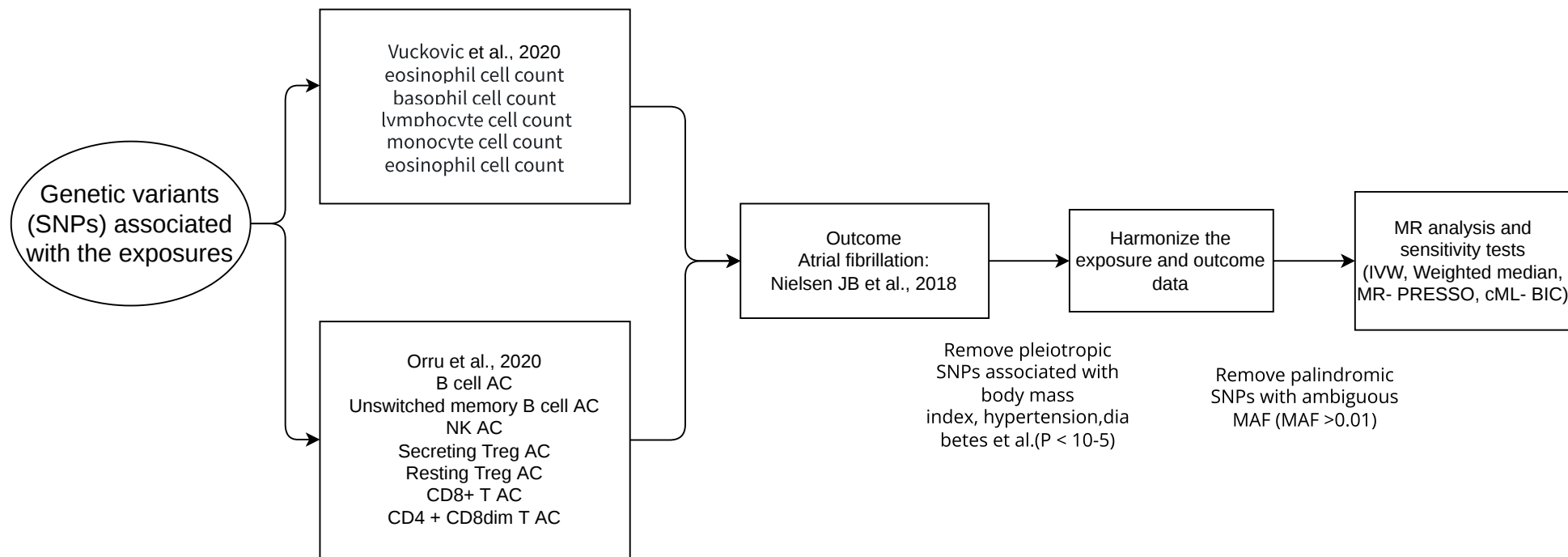

Supplement: Supplementary file 1 [file Data_Sheet_1.PDF]
